# Comprehensive Analysis of Long Non-Coding RNAs in Ovarian Cancer Reveals Global Patterns and Targeted DNA Amplification

**DOI:** 10.1371/journal.pone.0080306

**Published:** 2013-11-12

**Authors:** Rozita Akrami, Anders Jacobsen, Jessica Hoell, Nikolaus Schultz, Chris Sander, Erik Larsson

**Affiliations:** 1 Department of Medical Biochemistry & Cell Biology, Institute of Biomedicine, The Sahlgrenska Academy, University of Gothenburg, Gothenburg, Sweden; 2 Computational Biology Center, Memorial Sloan- Kettering Cancer Center, New York, New York, United States of America; 3 Department of Pediatric Oncology, Hematology and Clinical Immunology, Medical Faculty, Heinrich-Heine-University, Düsseldorf, Germany; Harvard School of Public Health, United States of America

## Abstract

Long non-coding RNAs (lncRNAs) are emerging as potent regulators of cell physiology, and recent studies highlight their role in tumor development. However, while established protein-coding oncogenes and tumor suppressors often display striking patterns of focal DNA copy-number alteration in tumors, similar evidence is largely lacking for lncRNAs. Here, we report on a genomic analysis of GENCODE lncRNAs in high-grade serous ovarian adenocarcinoma, based on The Cancer Genome Atlas (TCGA) molecular profiles. Using genomic copy-number data and deep coverage transcriptome sequencing, we derived dual copy-number and expression data for 10,419 lncRNAs across 407 primary tumors. We describe global correlations between lncRNA copy-number and expression, and associate established expression subtypes with distinct lncRNA signatures. By examining regions of focal copy-number change that lack protein-coding targets, we identified an intergenic lncRNA on chromosome 1, *OVAL*, that shows narrow focal genomic amplification in a subset of tumors. While weakly expressed in most tumors, focal amplification coincided with strong *OVAL* transcriptional activation. Screening of 16 other cancer types revealed similar patterns in serous endometrial carcinomas. This shows that intergenic lncRNAs can be specifically targeted by somatic copy-number amplification, suggestive of functional involvement in tumor initiation or progression. Our analysis provides testable hypotheses and paves the way for further study of lncRNAs based on TCGA and other large-scale cancer genomics datasets.

## Introduction

Recent transcriptomic studies in mammals have revealed an abundance of long non-coding RNAs (lncRNAs) that lie interspersed with coding genes in complex ways [1-3]. LncRNA transcripts typically have mRNA-like properties, such as multiexonic gene structures and poly(A) tails, but lack apparent protein-coding capacity. Although early functional examples (e.g. *H19* [4] and *XIST* [5]) were first described more than 20 years ago, lncRNAs are now emerging as widespread regulators of cell physiology with diverse roles both in the nucleus and the cytoplasm, including recruitment of histone-modifying complexes to chromatin, regulation of transcription and splicing, and control of mRNA translation.

Several recent studies suggest that lncRNAs may have important roles in oncogenesis [6,7]. For example, *HOTAIR* expression is high in metastatic breast cancer tumors, and its inhibition blocks metastasis in rodent models [8], *MALAT1* expression correlates with metastases and survival in lung cancer [9], and polyA+ transcriptome sequencing (RNA-seq) recently identified *PCAT-1* as a growth-promoting lncRNA in prostate cancer [10]. However, it is notable that the genetic data provided thus far primarily relates to alterations in gene expression. Malignant transformation requires genetic activation of growth-promoting oncogenes and inactivation of tumor suppressors, and this is facilitated in tumors by genomic instability, acquired genetic variability, and clonal expansion [11]. In large cancer genomics datasets, such as those produced by The Cancer Genome Atlas (TCGA) consortium, important cancer genes therefore reveal themselves through striking patterns of recurrent DNA-level alteration, including focal copy-number amplification and deletion [12,13]. However, while lncRNAs and coding genes should in principle be susceptible to activation or deactivation through similar mechanisms, there is thus far little evidence that lncRNAs are specifically targeted by copy-number alterations in cancer independently of proximal coding genes (recently reviewed in 6,14).

We here performed a large-scale genomic analysis of lncRNAs in high-grade serous ovarian carcinoma (HGS-OvCa), one of the leading causes of cancer death among women in the United States [15], based on high-throughput molecular profiles generated within TCGA [12]. We based our analyses on the comprehensive GENCODE lncRNA catalog, which has been subject to extensive characterization and manual curation [16,17], while using an annotation-unbiased approach where appropriate. We consequently focus on lncRNAs with reproducible expression in independent datasets, based on the assumption that cancer-relevant lncRNAs should, similar to coding genes, have important functions also in normal cells. Using deep coverage RNA-seq data and high-resolution DNA copy-number arrays, we derived simultaneous copy-number profiles and expression data for >10,000 GENCODE lncRNA genes across 407 primary tumors (data available at www.larssonlab.org/tcga-lncrnas). We investigate the global relationship between DNA copy-number and lncRNA expression, and evaluate lncRNAs in relation to established expression subtypes in HGS-OvCa. Moreover, we address whether lncRNAs may be specifically targeted by focal copy-number alteration in cancer.

## Results and Discussion

### Molecular profiling of lncRNAs in 407 tumors

**Figure 1:**
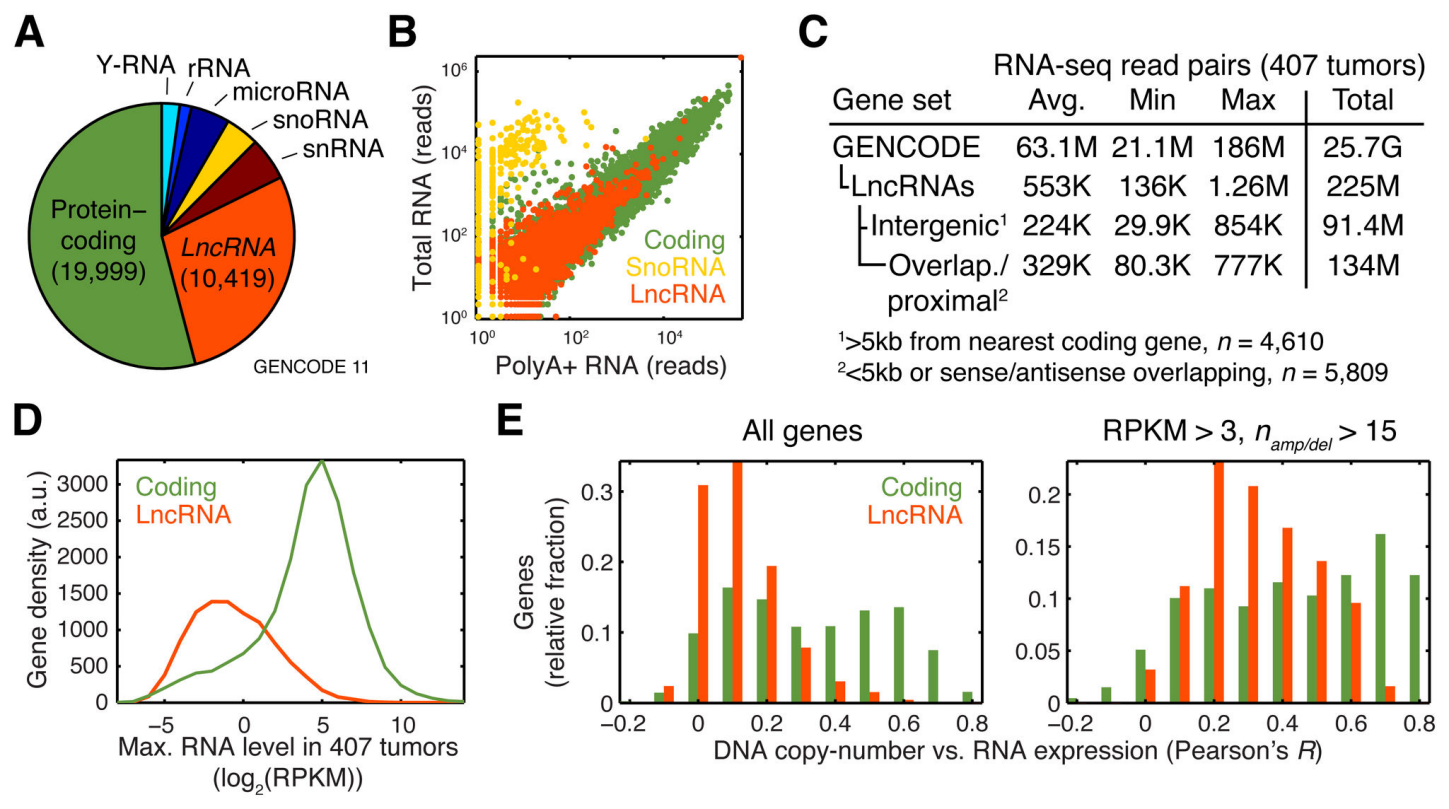
Molecular profiling of lncRNAs in 407 ovarian adenocarcinomas. **A**, Relative abundances of gene categories in the GENCODE 11 annotation (unique loci). **B**, Polyadenylation status of lncRNAs, determined by polyA+ vs. total RNA-seq from a mixture of 16 tissues. **C**, LncRNA expression profiling using polyA+ RNA-seq across 407 tumors. In total 25.7 billion uniquely mapped read pairs, encompassing >3 terabases, were counted in GENCODE genes. The table shows per-tumor sequencing depth, based on all GENCODE-mapped reads or lncRNA subsets. **D**, Distributions of lncRNA and coding gene expression levels (maximum RPKM in all tumors). RPKM, reads per kilobase per million reads. **E**, Histograms of correlations between DNA copy-number and RNA level (based on 407 tumors with dual data). Left panel: lncRNAs overall (left panel, *n* = 10,066), showing lower correlations compared to coding genes. Right panel: improved correlations when considering genes expressed at RPKM > 3 (top 19% lncRNAs, *n* = 1920) that were also amplified or deleted in >15 samples (right panel, *n* = 125).

We used the GENCODE [17] annotation as our main framework to investigate patterns of lncRNA copy-number alteration and expression across 407 stage-II-IV HGS-OvCa tumors [12]. We found that the GENCODE lncRNA subset [16], which encompasses 10,419 manually annotated lncRNA genes (version 11, Figure **1A**), showed a high degree of polyadenylation as determined by normal tissue RNA sequencing (RNA-seq) data (Figure **1B**). Copy-number data from comparative genomic hybridization (CGH) arrays was available for 486 primary tumors, and the vast majority of GENCODE lncRNAs (97%) expectedly resided in covered regions. We next processed a total of 25.7 billion GENCODE-mapped read pairs from polyA+ RNA-seq (on average 63.1 million per sample) to derive expression profiles for all GENCODE lncRNAs in 407 of these tumors (Figure **1C**). Only 225 million read pairs mapped to lncRNA loci (on average 553,000 per sample), emphasizing the need for high sequence coverage to accurately quantify lncRNAs.

Comparison of length-normalized (RPKM-type [18]) expression values between coding and lncRNAs genes confirmed that lncRNAs were expressed at substantially lower levels (Figure **1D**), in agreement with previous reports from a wide variety of cellular sources [1,16,19]. While 87% of coding genes showed an RPKM level >1 in at least one tumor, only 36% of lncRNAs reached this level of expression. Genome-wide correlations between DNA copy-number amplitude and RNA levels were lower for lncRNAs compared to coding genes, but this discrepancy was reduced when only considering abundant and frequently copy-number altered genes (Figure **1E**). A complementary analysis based on Affymetrix Exon 1.0ST arrays, which can interrogate a subset of lncRNAs, yielded similar results (481 samples, Figure **S1** in File S1).

### LncRNAs associate with expression subtypes

Previous analysis of coding gene expression in HGS-OvCa identified four robust subtypes, which were termed ‘immunoreactive’, ‘differentiated’, ‘proliferative’ and ‘mesenchymal’ based on their gene content [12,20]. The subtypes were further shown to be associated with specific genomic alterations, where the proliferative group has a lower frequency of *MYC* amplification and *RB1* deletion, while the immunoreactive group has a higher frequency of *MECOM* amplification. We here tested if established subtypes in HGS-OvCa also have distinct patterns of lncRNA expression.

**Figure 2:**
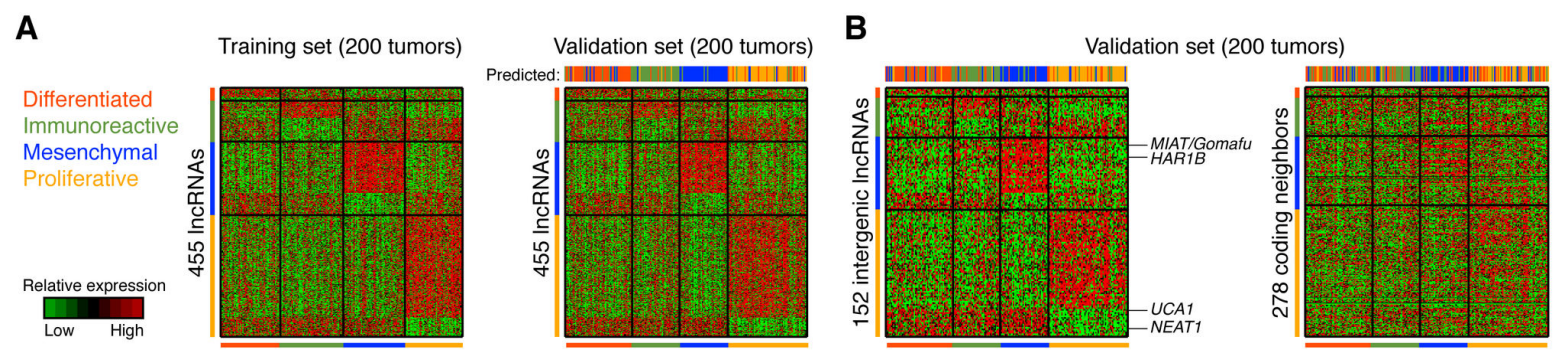
LncRNAs associate with expression subtypes. **A**, 455 lncRNAs showed increased or reduced expression in one of four previously defined expression subtypes (200 random tumors, left). These lncRNAs maintained their subtype-selective expression patterns in 200 independent tumors, and subtype could there be predicted based on their expression at 77% accuracy (right). **B**, The same analysis based on an intergenic lncRNA subset (*n* = 152, 73% accuracy, left). Nearest up- and downstream protein-coding neighbors of these lncRNAs (278 unique genes) lacked strong subtype-specific expression patterns and their combined signature was less informative of subtype (51% accuracy, right).

Tumors with available subtype, clinical and expression data were randomly partitioned into two sets (*n* = 200 each), withholding one half for later validation. We identified 455 lncRNAs that were induced or repressed specifically in one of the four subtypes relative to remaining samples (Figure **2A**, as detailed in Methods). These expression patterns were clearly maintained in the validation set, confirming that subtype associations were non-random (Figure **2B**). In addition, a tumor’s expression subtype could be predicted based on subtype-associated lncRNAs using a simple classifier (**Methods**) in the majority (77%) of tumors (Figure **2B**).

Antisense-overlapping lncRNA may show strong positive correlations with their coding hosts [16], motivating analysis based on intergenic lncRNAs alone. We therefore investigated a subset of 152 lncRNAs with intergenic localization, and found their expression patterns to be similar to the full set in the validation data (Figure **2B**, Table **S1** in File S1, 73% accuracy). Although coding neighbor genes of the 152 lncRNAs were still moderately predictive of subtype (51%), they showed considerably weaker patterns of subtype-specific expression (Figure **2B**). Notably, among intergenic lncRNAs induced in the mesenchymal subtype were *MIAT*/Gomafu, a known target and co-activator of Oct4 with a role in stem cell pluripotency [21]. *NEAT1* and *UCA1* were repressed in the proliferative subtype: *NEAT1* is essential for the structure of nuclear paraspeckles [22] and has been shown to be upregulated in ovarian cancer [23], while *UCA1* is a known regulator of cell growth in bladder carcinoma [24]. We additionally assessed lncRNA levels in relation to patient survival, but did not find reproducible associations. Our results show that expression subtypes in HGS-OvCa, originally defined based on coding gene expression, are each associated with distinct lncRNA expression signatures, and we speculate that these lncRNAs could contribute to transcriptional reprogramming or otherwise act in the cellular circuits that are altered in these tumor types.

### LncRNAs in regions of focal copy-number alteration

Tumor genomes are mosaics of chromosomal aberrations, of which some are under selection to activate or inactivate specific oncogenes or tumor suppressors. In large patient cohorts, individual targeted genes may therefore be deduced through patterns of recurrence, in particular when altered regions are narrow (focal) [25]. The GISTIC algorithm, when applied to copy-number data from TCGA ovarian cancer, identified several regions of focal recurrent copy-number alteration [12]. Many of these encompass well-known cancer genes, but in some cases the targets remain poorly defined. We hypothesized that lncRNAs could be drivers in some of these events, and therefore screened narrow focal regions for overlaps with lncRNAs.

**Figure 3:**
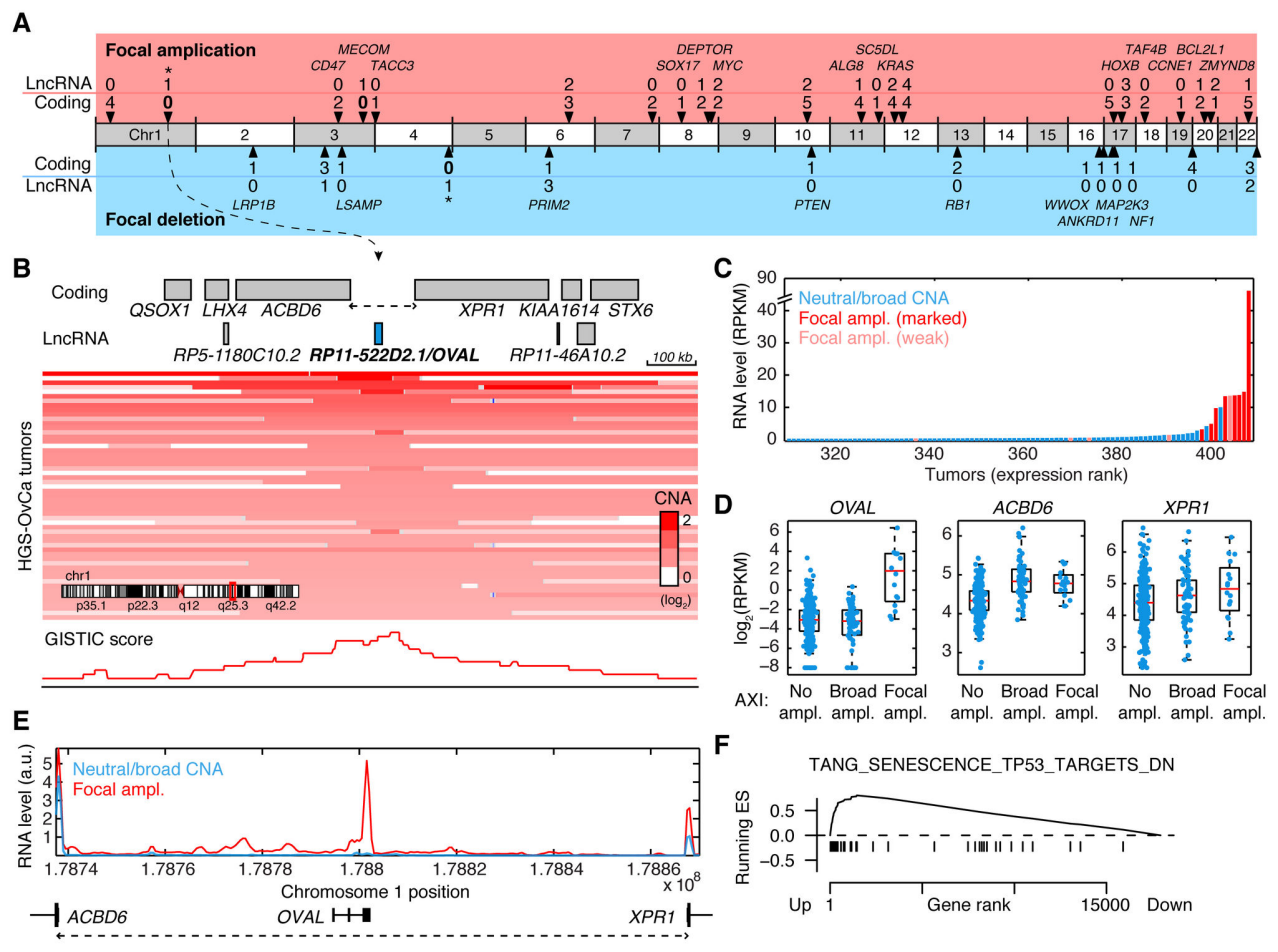
Recurrent lncRNA amplification in serous ovarian carcinoma. **A**, LncRNAs and coding genes in narrow regions of recurrent amplification or deletion identified by GISTIC (*q* < 0.05) in 486 HGS-OvCa tumors. Gene counts for 35 tight focal peaks with a maximum of 5 overlapping coding genes are shown. Known cancer genes and unambiguous coding targets are indicated. *, regions investigated in more detail. **B**, Detailed view of the *ACBD6*-*XPR1* intergenic region (AXI region, dotted line) in a ~1 Mb genomic context. The AXI focal peak is centered on *RP11-522D2.1/OVAL*, an uncharacterized lncRNA on chr1q25. Red shading shows copy-number profiles for individual tumors. **C**, Tumors ordered by *OVAL* expression. *OVAL* RNA (y-axis) was mostly low or undetectable, but was induced in focally amplified cases (as defined in Methods). **D**, Neither *ACBD6* nor *XPR1* were notably induced by AXI region focal amplification. *OVAL* RNA was low also in broadly amplified cases. ND, not detected. **E**, Average RNA-seq read density in the AXI region (dotted line) for tumors with marked AXI focal amplification (*n* = 10) compared to remaining tumors (normalized read counts per 1000 nt segment). **F**, GSEA analysis showed than experimentally determined P53 regulated genes are induced in *OVAL* focally amplified tumors.

There were 35 narrow (overlapping with at most 5 coding genes) amplifications or deletions that were significant at a false discovery rate (residual *q*) of 0.05 (Figure **3A**, Table **S2** in File S1). Many overlapped with established proto-oncogenes such as *CCNE1* and *MYC*, and the *RB1*, *NF1* and *PTEN* tumor suppressors, but lncRNAs were also present together with coding genes in several cases (Figure **3A**). Although selection for copy-number alteration at these loci could in principle be explained by lncRNAs, either alone or in combination with their coding neighbors [26], we focused instead on two focal peaks that lacked protein-coding genes: an amplification on 1q25 and a deletion on chromosome 4q34 (indicated with * in Figure **3A**). The deleted region was in a large intergenic space ~1 Mb from *ODZ3*; a gene recently found to be targeted by L1 retrotransposition in colorectal cancer [27] and deleted in neuroblastoma [28]. While deleted segments in HGS-OvCa were clearly separated from *ODZ3* and encompassed two annotated lncRNA genes (Figure **S2A** in File S1), these lacked relevant expression (<5 mapped reads in >99% of tumors), and we failed to reveal other candidates by investigating RNA-seq read coverage in the region (data not shown). Further analysis suggested that the deletions might be indirectly targeted at *ODZ3* through disruption of associated regulatory DNA (Figure **S2B** in File S1), and the region was not further characterized.

### Focal somatic amplification of OVAL lncRNA

We next investigated the 1q25 amplification, which was focally gained in 16/407 patients (3.9%), and centered on a 128 kb intergenic region between the *ACBD6* and *XPR1* genes (henceforth the AXI region). The AXI region lacks protein-coding genes, but contains a single annotated lncRNA gene, *RP11-522D2.1*, near its center (Figure **3B**). This lncRNA, which we here term *OVAL* (ovarian adenocarcinoma amplified lncRNA), coincides closely with the focal peak identified by GISTIC, and is positioned 55 kb and 65 kb from the nearest coding protein-coding neighbors. Notably, the amplified chromosomal segments were often small (50-100 kb) and encompassed the full *OVAL* gene, while being constrained to the AXI region or extending only partially into the neighboring genes (Figure **3B**).

Since the focal DNA amplification pattern pointed to *OVAL* as the alteration-driving gene in this region, we next investigated if this was supported by the expression pattern of *OVAL* in the tumors. *OVAL* expression was low or absent in both normal fallopian tube (Figure **S3** in File S1) and in the majority of tumors, including most cases with wide 1q amplification. However, focal amplification of the *OVAL* locus coincided strikingly with *OVAL* transcriptional activation (Figure **3C**). *OVAL* RNA was on average 46-fold higher in focal cases compared to remaining samples (*P* = 3.5e-8, Wilcoxon rank sum test), and *OVAL* ranked 74th of all GENCODE lncRNAs based on maximum expression in all tumors (Table S3 in File S1). Similar results were obtained using hybridization-based Exon 1.0ST data (Figure S4 in File S1).

Although AXI region focal amplification did not appear to directly target the flanking coding genes, these could still be indirectly affected at the level of gene expression. This would be compatible with their regulatory sequences being altered, or *OVAL* having a *cis*-regulatory role in controlling their transcription. However, neither *ACBD6* nor *XPR1* were notably induced in focally amplified cases (Figure **3D**). In addition, these genes are not previously described as altered in cancer, further supporting that *OVAL* is independently targeted in the AXI intergenic region.

Investigation of RNA-seq read coverage in the AXI region revealed that *OVAL* was the main expressed locus in focally amplified tumors, while remaining samples showed low transcriptional activity in this region (Figure **3E**). Although additional transcription was observed outside of the *OVAL* locus, most notably in the upstream region (Figure **3E**), these signals were not consistent between individual tumors (Figure S5 in File S1). Examination of available data in Genbank revealed only a few singular ESTs sequences in the AXI region away from the focal center, while *RP11-522D2*.*1*/*OVAL* was supported by 12 spliced ESTs and 6 cDNA sequences. A putative Y-RNA (predicted from RFAM families), close to the AXI region edge 50 kb from the focal peak, was not supported by cDNA/EST evidence or RNA-seq in tumors or normal tissue (data not shown). We conclude that both HGS-OvCa tumor expression profiles and available cDNA/EST evidence point to *OVAL* as the main stably transcribed unit in the amplified AXI region.

Gene set enrichment analysis (GSEA) revealed that previously defined targets of P53 (TANG_SENESCENCE_TP53_TARGETS_DN) were significantly elevated in *OVAL* amplified tumors (Figure **3F**). While this indicates that *OVAL* activation may coincide with altered P53 activity, *TP53* mutation status and mRNA levels were similar in both groups. Genes encoding muscle-related contractile proteins (STRUCTURAL_CONSTITUENT_OF_MUSCLE) were enriched among those repressed in *OVAL* amplified tumors.

### Molecular characterization of OVAL

**Figure 4:**
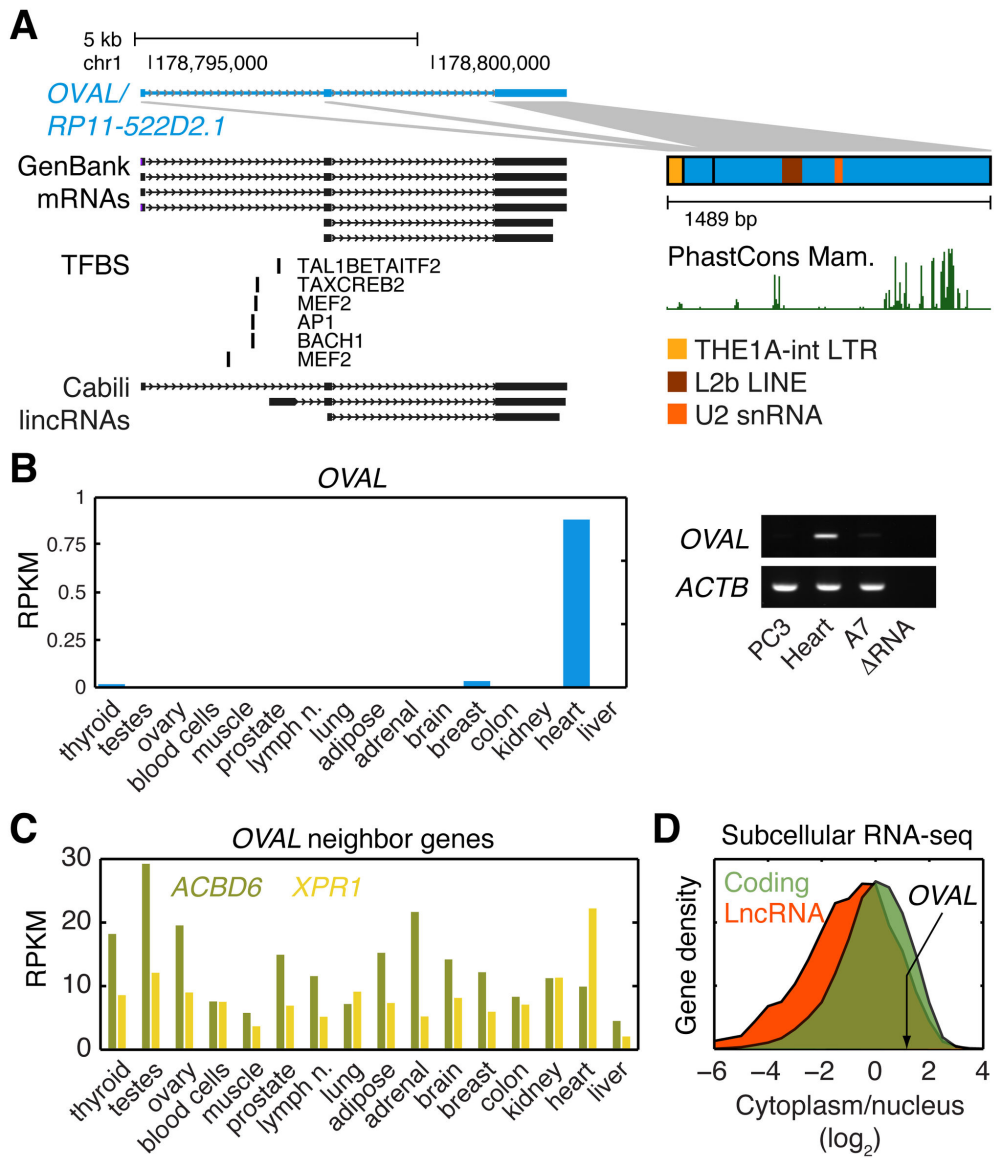
Properties of *OVAL* lncRNA. **A**, *OVAL* locus on chromosome 1. GenBank mRNAs, conserved transcription factor binding sites (TFBS) predicted by the UCSC brower, and other features are indicated. **B**, Normal tissue expression profile of *OVAL* (RNA-seq). Heart expression was confirmed by reverse transcription PCR. PC3, human prostate cancer cell line; Heart, heart auricle; A7, human melanoma cell line **C**, *OVAL* and its coding neighbors have disparate expression profiles. **D**, Subcellular RNA-seq from 7 pooled cell lines (Gm12878, HelaS3, HepG2, Huvec, H1hesc, Nhek and K562) shows predominant cytoplasmic localization.

Having established *OVAL* as a likely target in the AXI region, we further characterized this gene in terms of gene structure and normal tissue expression. The *OVAL* gene contains three annotated exons that give rise to a predicted 1489 nt non-coding RNA, where the large third exon contributes most of the sequence. This structure was supported by multiple GenBank mRNA sequences (Figure **4A**) and spliced ESTs, as well as RNA-seq from cell lines such as Gm12878 (data not shown). Many of the *OVAL* ESTs and mRNAs originated from human melanoma cells, and the transcript was consequently pinpointed in a recent bioinformatics screen for melanoma-specific public ESTs [29]. *OVAL* was also mapped in a recent survey of human intergenic lncRNAs [19], and similar to our own analysis of normal tissue RNA-seq data, this study identified an alternative first exon isoform (Figure **4A**) not supported by the tumor expression data. Although additional possible splicing patterns were observed in the tumors, these showed weak and inconsistent expression across samples (data not shown).

Evolutionary conservation, based on a mammalian genomic multiple alignment, was low overall, but specific regions in the last exon demonstrated elevated conservation (Figure **4A**). The miRcode database of putative microRNA target sites in lncRNAs [30] revealed a conserved miR-30 site, present in most primates and mammals, in one of these patches. Although the mature sequence is mainly non-repetitive, RepeatMasker [31] identified THE1A-int LTR and L2b LINE derived elements, as well as a possible U2 snRNA sequence in the last exon (Figure **4A**). However, we found no matches to snRNAs or other known structures in RFAM [32], and *OVAL* is therefore unlikely to function as a precursor for a classical structural RNA.

LncRNAs have previously been defined on the basis of codon substitution frequency scores and the lack of an open reading frame (ORF) larger than 100 amino acids [1]. In addition to being classified as non-coding by the GENCODE pipeline, the mature *OVAL* sequence was non-coding according to the CPC algorithm [33] and using PhyloCSF [34] based on a mammalian alignment. ORFs in *OVAL* all lack the Kozak consensus and are no longer than 98 amino acids. A recent joint analysis of tandem mass spectrometry data and GENCODE lncRNA sequences, including *RP11522-D2*.*1*/*OVAL*, identified only one single lncRNA match when excluding misannotated cases, supporting an overall lack of coding capacity for these transcripts [35]. Importantly, no homology to any known protein sequence was revealed by BLASTx analysis. A coding function thus appears improbable, and we conclude that *OVAL* likely represents a *bona fide* lncRNA.

RNA-seq from normal human tissues showed that *OVAL* is selectively expressed in heart muscle, and this was confirmed by reverse transcription PCR (Figure **4B**). Two putative conserved myocyte enhancer factor-2 (MEF2) binding sites, positioned close to the alternative first exon (Figure **4A**), may drive expression in muscle tissue, as both heart muscle and human skeletal muscle myoblast (HSMM) cells exclusively express this alternative isoform (data not shown). The *OVAL* expression pattern is markedly different from its coding neighbors (Figure **4C**), and its subcellular localization was predominantly cytoplasmic (Figure **4D**). This further speaks against a *cis*-regulatory role on nearby genes, and is consistent with our finding that *OVAL* amplification did not notably influence their expression (Figure **4D**). In summary, *OVAL* appears to have a cytoplasmic non-coding function that is independent of its protein-coding neighbors.

### OVAL amplification in serous endometrial carcinomas

**Figure 5:**
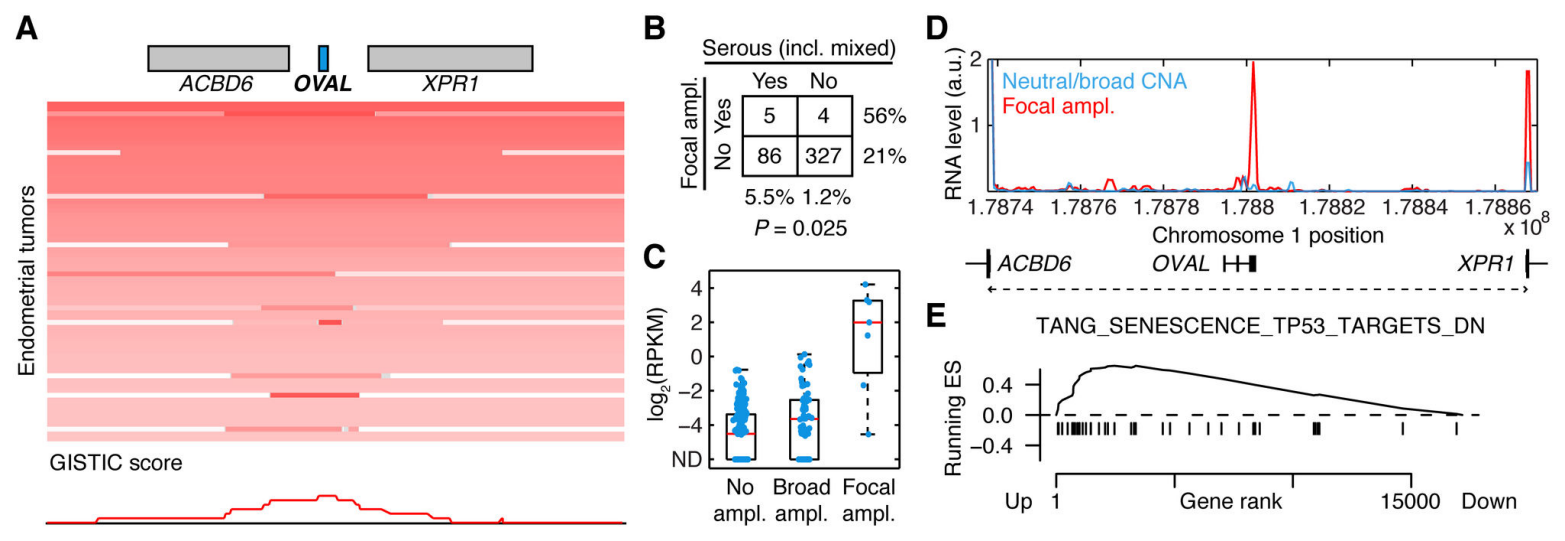
The *OVAL* locus is focally amplified in serous endometrial tumors. **A**, Low frequency focal amplification of the *OVAL* locus in endometrial cancer, but not 16 other TCGA cancers (see Figure **S6** in File S1). **B**, 56% of focally amplified cases were of the serous subtype, compared to 21% overall (*P* = 0.025, Fisher’s exact test). **C**, *OVAL* RNA was strongly induced in a subset of tumors, and this coincided with focal amplification of the AXI region. ND, not detected. **D**, Average RNA-seq read density in the AXI region for tumors with marked focal amplification (*n* = 4) compared to remaining tumors (normalized read counts per 1000 nt segment). **E**, Similar to ovarian cancer, GSEA analysis revealed induction of P53 targets in *OVAL* amplified tumors.

We next investigated whether *OVAL* amplification is unique to ovarian cancer, and considered copy-number profiles from 16 additional TCGA cancers, ranging in size from 57 to 825 tumors. Interestingly, we observed low-frequency focal amplification of the *OVAL* locus also in uterine corpus endometroid carcinoma, while no obvious focal signal was seen in the remaining cancers (Figure S6 in File S1). Closer inspection revealed that the focal peak again coincided closely with the *OVAL* gene (Figure **5A**).

A fraction of endometrial tumors are classified as serous or serous-like. These have a close morphological resemblance to their ovarian counterpart [36], and are also genetically similar to ovarian cancer [37]. Consequently, we observed that tumors of the serous subtype were >4 times as likely to carry *OVAL* focal amplification compared to non-serous tumors (5/91 vs. 4/331, *P* = 0.025, Figure **5B**). Similar to ovarian cancer, focal amplification of the AXI region was associated with strongly increased expression of *OVAL* (Figure **5C**), and RNA-seq read coverage showed that *OVAL* was the main transcribed unit in the region (Figure **5D**). GSEA analysis revealed that experimentally determined P53 regulated genes were upregulated in *OVAL* amplified samples, replicating our previous results from ovarian cancer (Figure **5E**). Taken together, results from ovarian and endometrial cancer suggest that *OVAL* amplification is selected for specifically in serous tumors irrespective of tumor site.

## Conclusions

LncRNAs have previously been assayed in clinical materials using next generation sequencing, including a recent study of 64 carcinomas and sarcomas using 3’ end sequencing [38], and transcriptome sequencing of 102 prostate tissues and cell lines [10]. In addition, lncRNAs were profiled in normal and cancer tissues based on 272 public SAGE libraries [39]. The present analysis is the first to make use of TCGA RNA-seq to profile lncRNAs in cancer, and to facilitate future investigation we make lncRNA molecular profiles for TCGA tumors available at www.larssonlab.org/tcga-lncrnas.

There is only limited evidence for somatic focal copy-number alteration of lncRNAs in cancer, and described cases involve lncRNAs that are co-altered with proximal coding cancer genes. Two lncRNAs in the *LSAMP* tumor suppressor locus on chromosome 3q13, *OC285194* and *BC040587*, were frequently focally deleted in osteosarcoma, often together with *LSAMP* [26]. These lncRNAs are coexpressed with *LSAMP*, and the three genes are likely functionally interconnected. The *PVT1* locus on 8q24, which gives rise to a variety of spliced non-coding RNAs, is often co-amplified with the nearby *MYC* oncogene [40,41]. However, in time it has become clear that *PVT1* encodes several microRNAs, and its primary role could therefore be that of a microRNA precursor [42,43].

In the case of *RP11/522D2.1/OVAL*, several independent observations nominate it as an independent target of somatic gene amplification. It is located at the center of a narrowly amplified intergenic segment that lacks other annotated genes, and the focal peak closely coincides with the *OVAL* gene. RNA-seq read coverage, as well as available cDNA and EST evidence, failed to reveal other credible candidates in the region. Focal, but not broad, amplification coincided with strong induction of *OVAL* RNA. *OVAL* was not co-expressed with its coding neighbors, none of which are previously associated with cancer and the closest being more than 50 kb away, and *OVAL* amplification did not notably alter their expression. This, in combination with a predominantly cytoplasmic localization, speaks against *OVAL* having a *cis*-regulatory role on neighbor genes. The replication of these patterns in serous endometrial cancer reinforces the hypothesis.

Focal amplification of the AXI region is relatively rare (3.9%), and amplitude gains typically low. However, HGS-OvCa is characterized by great mutational diversity, with a relative lack of single frequent driving alterations, and the frequency is comparable to known functional alterations such as BRCA1 and BRCA2 somatic mutation (3.5% and 3.2%, respectively [44]). Even single-copy gains in the AXI region were associated with strong induction of *OVAL* RNA, similar to e.g. *IGF2* in colorectal cancer [13]. This implies that copy-number gain coincides with non-copy-number mechanisms to activate transcription in these tumors. Several recent studies point to cytoplasmic roles for lncRNAs, including posttranscriptional regulation by complementary base pairing with mRNAs [45,46] and inhibitory binding to microRNAs [47-49]. Future experimental studies are needed to unravel the putative cytoplasmic function of *OVAL* RNA, and to understand how its hyperactivation may contribute to serous tumor development.

LncRNAs have until now received little attention in large-scale cancer genomics efforts such as TCGA. This report complements the coding-centric framework used for the original TCGA HGS-OvCa analysis, and paves the way for futures studies of lncRNAs based on these powerful datasets.

## Methods

### GENCODE annotation and lncRNA definition

Tab-delimited files and BED files, describing the GENCODE V11 gene annotation, were obtained through the UCSC browser (the most current release available on May 11 2012 for the hg19 assembly). GENCODE ‘comprehensive’ and ‘pseudogene’ sets were merged, and transcripts with ambiguous genome mapping were removed (*n* = 330), resulting in a set of 179,526/53,433 transcripts/genes with unique mapping to a single locus. To define a lncRNA subset, we relied on the coding/non-coding classification provided by the GENCODE/ENSEMBL pipeline, and considered as lncRNAs genes that exclusively produce transcripts of the ‘antisense’, ‘lincRNA’, ‘non_coding’ and ‘processed_transcript’ types. Genes producing non-coding mature transcripts shorter than 200 nt were excluded, as were genes with symbols matching any coding genes in the RefSeq or UCSC xenoRefGene set. This removed a small number of cases in GENCODE of obvious incorrect coding/non-coding classification, and resulted in a final set of 15,977/10,419 lncRNA transcripts/genes. An ‘intergenic’ lncRNA subset was further defined by determining, for each gene, the smallest distance from either the 5’-most transcript start or the 3’-most transcript end to the nearest coding GENCODE gene, and requiring this distance to be >5 kb.

### Genomic copy-number data

We used segmented genomic copy-number data for 486 unique patients, produced on the Agilent 1M platform and segmented using binary circular segmentation as described in the original TCGA ovarian study [12]. As these were generated using the Hg18 genome assembly as reference, we used the LiftOver tool (http://genome.ucsc.edu/util.html) to remap the GENCODE gene annotation from Hg19 to Hg18. Genes were assigned copy-number amplitudes (log_2_ scale) by comparing gene coordinates with segment coordinates to identifying overlapping segments. In cases of partial overlap with several segments the minimum amplitude was chosen, the rationale being that amplification of the complete gene would normally be required to increase its activity, while partial deletion would normally disrupt its function. Genes that had undetermined copy-number amplitudes in more than 50% of samples were excluded, resulting in a final copy-number matrix covering 53,433 genes of which 10,066 were lncRNAs, 11,316 were pseudogenes and 19,061 were protein-coding. Segmented copy-number data for endometrial tumors (*n* = 443, Hg19 assembly), produced on the Affymetrix SNP6 platform, were obtained through the TCGA data matrix and processed using the same pipeline.

### TCGA RNA-seq processing

RNA-seq sequence libraries in BAM format (2x75 nt paired-end reads) for 412 primary HGS-OvCa tumors were downloaded from cgHub (http://cghub.ucsc.edu, data available on Oct 9 2012). The BAM files were produced by the BCCA Genome Science Center TCGA RNA-seq pipeline, which briefly uses BWA [50] for alignment to the Hg18 genome assembly and to exon junctions derived from Ensembl/GENCODE, UCSC genes and RefSeq. Low-quality alignments (mapping quality 0) were removed and sequences were name-sorted and converted to SAM format using SAMtools [51]. We used TopHat [52] with default parameters to realign a subset of the samples to enable unbiased study of splicing patterns in the AXI region. TCGA endometrial RNA-seq data in BAM format (76 nt single-end reads) for 321 tumors was obtained from cgHub (downloaded on Oct 18 2012). These BAM files are not directly useful for quantifying GENCODE lncRNAs as they were generated by alignment to a limited transcriptome database. They were therefore converted to FASTQ format and realigned to the Hg19 assembly with TopHat using the “-G” option with known splice junctions from GENCODE. Read counts for individual GENCODE genes were subsequently determined using HTSeq-count (http://www-huber.embl.de/users/anders/HTSeq) in “intersection-strict” mode, by considering only uniquely mapped reads. RPKM expression levels for lncRNAs (*n* = 10,419) and other GENCODE genes were finally calculated by normalizing for mRNA length and library size as determined by the number of GENCODE-mapped reads. For analyses requiring log_2_-scale values, a pseudo value of 0.01 was added before conversion to avoid log of zero [53]. HGS-OvCa samples with less than 20 million GENCODE-mapped read pairs and without matching copy-number data were excluded, resulting in a final set of 407 tumor expression profiles with on average 63.1 million GENCODE-mapped read pairs each (25.7 billion in total). For endometrial samples, 10 million GENCODE-mapped reads were required, for a resulting final set of 293 tumors with on average 19.1 million GENCODE-mapped reads (5.6 billion in total). Expression coverage plots were generated by dividing the genome into partially overlapping 1000 nt tiles spaced 500 nt apart. Tile read counts were determined using BEDTools (coverageBed utility) [54], and these were normalized based on the median of the top 5% expressed tiles in each sample.

### Exon array processing

565 Affymetrix Human Exon 1.0ST array (HuEx) CEL files for primary HGS-OvCa tumors were obtained from the TCGA (level 1 data). Probeset intensities (1,411,399 probesets) were determined using Affymetrix APT software suite using the RMA-sketch algorithm. Genomic probeset locations (Hg19 assembly) for the HuEx array were obtained from Affymetrix, and subsequently mapped to exons in our GENCODE-derived gene annotation. For each gene, all exonic probeset signals (linear scale) were averaged to produce a single gene expression value. The final expression matrix contained expression levels for 45,953 genes of which 8,777 were lncRNAs, 9,902 were pseudogenes and 19,774 were protein-coding.

### Screening for lncRNAs in focal regions

Focally amplified or deleted genomic loci, identified by the GISTIC algorithm [25] as described previously [12], were screened for overlaps with annotated lncRNAs. We considered the “wide peak” as defined by GISTIC, and emphasized those that contained annotated lncRNAs while lacking coding genes. For integrative analyses requiring cases of OVAL/AXI region focal amplification to be defined, we choose the following rules: Tumors were classified as focal if the difference between the AXI center copy-number amplitude (CNA) and the minimum CNA in the upstream *ACBC6* region (log_2_ scale), as well as the downstream *XPR1* region, was >0.2, or alternatively >0.4 (marked focal amplification). A CNA threshold of 0.4 was used to define broadly amplified samples. To investigate putative focal amplification of the *OVAL* locus in other cancers than ovarian, GISTIC results for additional TCGA cancers were obtained from the Broad Institute Firehose pipeline. We considered 16 additional cancers where copy-number data was available for at least 50 patients (ranging from 57 to 825) at the time of download (Sept 19, 2012). Amplified segments and GISTIC scores were visualized with IGV.

### Additional RNA-seq analyses

Raw reads from the Illumina BodyMap2 RNA-seq expression compendium, including data from 16 individual normal human tissues (2x50 nt, unstranded), as well pooled RNA from the same tissues (100 nt, stranded), was obtained from EBI’s ArrayExpress repository (accession E-MTAB-513). To determine the polyA status of lncRNAs, sequence traces from DSN-normalized pooled total RNA (148 million reads) and from DSN-normalized polyA+ pooled RNA (287 million reads) were mapped to the Hg19 assembly using TopHat and quantified using HtSeq-count as described above for TCGA datasets, after stripping of adapter sequences using the FastX toolkit (http://hannonlab.cshl.edu/fastx_toolkit). Tissue expression profiles were determined by applying a similar workflow on reads from the 16 tissues (2.54 billion reads in total).

To determine the subcellar localization of *OVAL*, subcellular RNA-seq reads produced within the ENCODE project [55] were obtained through the UCSC FTP server. A total of 1.71 billion 76 nt read pairs from polyA+ nuclear and cytoplasmic fractions from seven human cell lines (Gm12878, HelaS3, HepG2, Huvec, H1hesc, Nhek and K562) were aligned to the human hg19 reference genome with Tophat. Ratios between pooled normalized read counts from nuclear and cytoplasmic fractions were finally calculated for all GENCODE genes.

### Subtype and gene set enrichment analyses

We used the Matlab CGDS toolbox [44] to access TCGA subtype data within the Matlab (Mathworks Inc.) environment. Subtype-specific lncRNAs were identified using the *t*-statistic based on log-scale expression values, by testing for differential expression between tumors in one subtype and remaining tumors. Genes with more than 50% undetermined values (0 reads) were excluded. A *t* threshold of 4/-4 was applied to find subtype-associated lncRNAs, and lncRNAs that were ambiguously assigned to more than one subtype were not considered. Log-scale expression values for each gene were z-score normalized prior to visualization. Prediction of subtypes from lncRNA expression profiles was based on a simple scoring system, where the difference between the means of z-score normalized expression values of previously defined subtype-induced and sub-type repressed lncRNAs was calculated. This was done separately for each subtype, choosing the highest scoring subtype in each tumor.

We used GSEA [56] to identify differentially expressed gene sets in the tumor samples with focal *OVAL* amplification. We quantified differential expression between two phenotypes (amplification vs. rest) using the t-statistic, and considered all gene sets (MSigDB) of size 15-500 genes (*n* = 5332 gene sets). Gene set enrichment *P*-values were computed with respect to a null distribution obtained from 1000 randomizations of the patient-phenotype labels.

### Reverse transcription PCR

RNA from heart auricle was a kind gift from Dr. Elin Stenfeldt, and RNA from A7 melanoma cells (ATCC) and PC3 prostate cancer cells [57] were kindly provided by Dr. Levent Akyurek. Total RNA was reverse-transcribed using the High Capacity cDNA kit (Applied Biosystems). PCR (55 annealing temperature, 35/30 cycles for OVAL/ACTB) was performed using the following primers: RP11-522D2.1/*OVAL*: 5’-AGGCCAATATGCAGACAAGG-3’ and 5’-AGTTCTCCAGTGGGGGTCTT-3’; *ACTB*: 5’- ACTCTTCCAGCCTTCCTTCC-3’ and 5’-GTACTTGCGCTCAGGAGGAG-3’.

## Supporting Information

File S1 Supplementary figures and tables.
(PDF)
