# Supplementary material for: Comprehensive Analysis of Long Non-Coding RNAs in Ovarian Cancer Reveals Global Patterns and Targeted DNA Amplification
Source: PLoS One. 2013 Nov 12;8(11):e80306. doi: 10.1371/journal.pone.0080306 (PMC3827191; doi:10.1371/journal.pone.0080306)
Supplement: File S1 — Supplementary figures and tables. (PDF) [file pone.0080306.s001.pdf]

SUPPORTING INFORMATION

Supporting figures

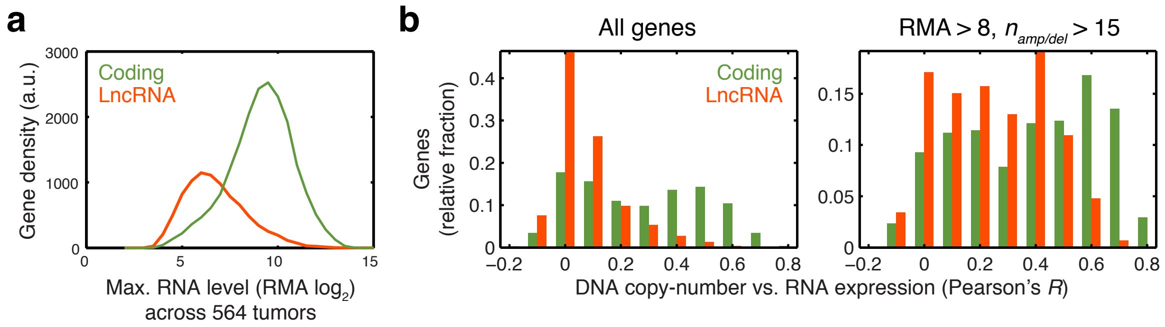

Supplementary Figure 1. Global patterns of lncRNA expression vs. copy-number variation in ovarian adenocarcinomas based on HuEx 1.0ST expression arrays. a, Distributions of lncRNA and coding gene expression levels (maximum signal in all tumors, as determined by the Affymetrix RMA algorithm). **b,** Histograms of correlations between DNA copy-number and RNA level (based on 481 tumors with dual data). Left panel: lncRNAs overall (left panel, *n* = 8,478), showing lower correlations compared to coding genes. Right panel: improved correlations when considering genes expressed at RMA level > 8 (top 21% lncRNAs, *n* = 1754) that were also amplified or deleted in >15 samples (right panel, *n* = 146).

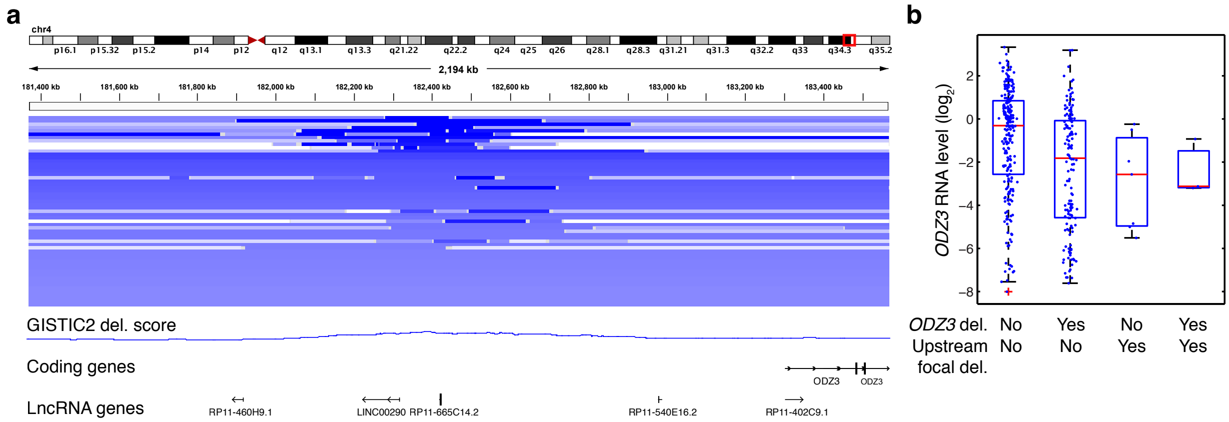

Supplementary Figure 2. Focal deletion on 4q34.2. a, An intergenic focally deleted region, pinpointed by GISTIC analysis, harbors lncRNAs RP11-665C14.2 and, slightly off-center, LINC00290. However, RNA-seq data did not reveal convincing expression of these genes or other candidates. **b,** Focal deletions are associated with reduced expression of the downstream *ODZ3* gene, even when *ODZ3* is not by itself copy-number reduced (a $\log_2$ threshold of -0.4 was here used to define deleted samples). This is compatible with the deleted segments being part of a regulatory region required for *ODZ3* transcriptional activation, acting at ~1 Mb distance.

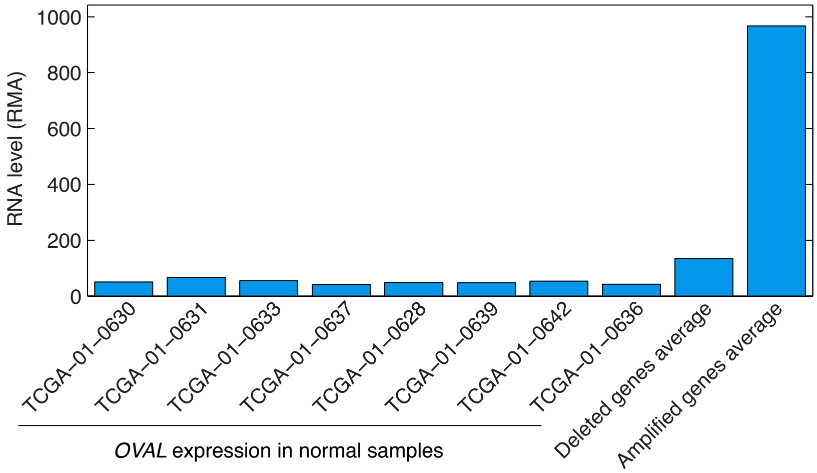

Supplementary Figure 3. *OVAL* expression in normal samples. *OVAL* expression was assayed in 8 normal fallopian tube samples using TCGA Affymetrix Exon 1.0ST data. The average linear RMA value was 50.8, to be compared with 134.0 for homozygously deleted genes ($\log_2$ CNA < -3) and 967.4 for amplified genes ($\log_2$ CNA > 3).

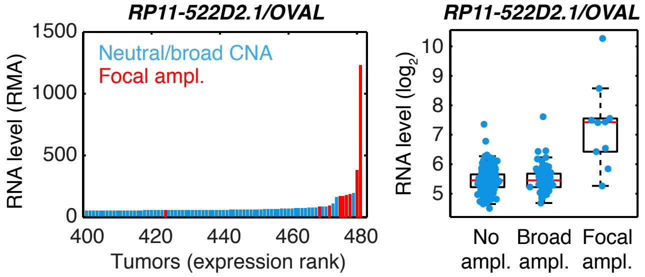

Supplementary Figure 4. OVAL focal amplification correlates with transcriptional activation. Affymetrix Exon 1.0ST data mirrors results obtained using RNA-seq: *OVAL* RNA is low or undetectable in most tumors, and induced specifically in focally amplified cases.

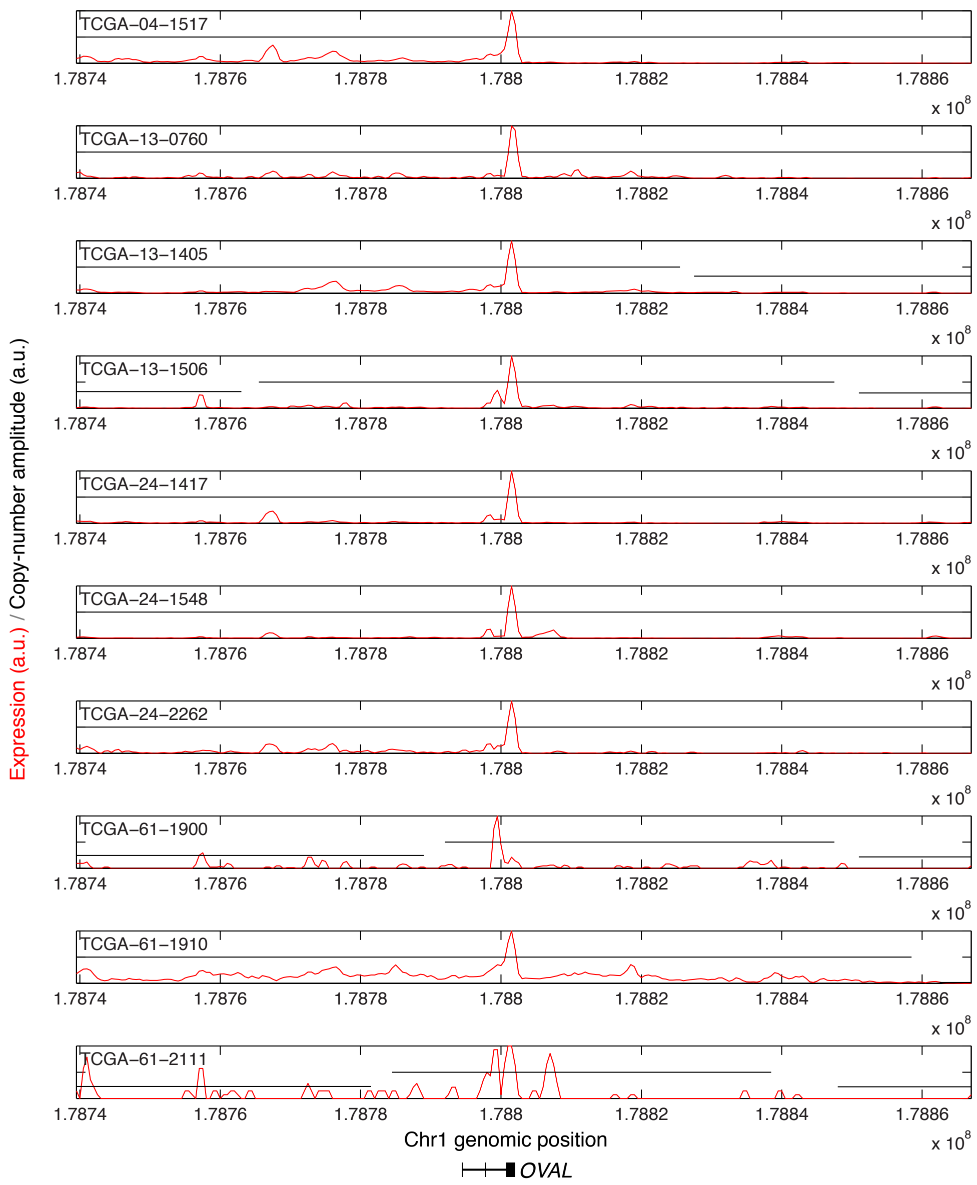

Supplementary Figure 5. Read density and copy-number amplitude in AXI region focally amplified samples. The plots show normalized read density (based on reads counts aggregated per 1000 nt segment) and copy-number patterns in tumors with marked AXI focal amplification (relative CNA > 0.4 as defined in Methods).

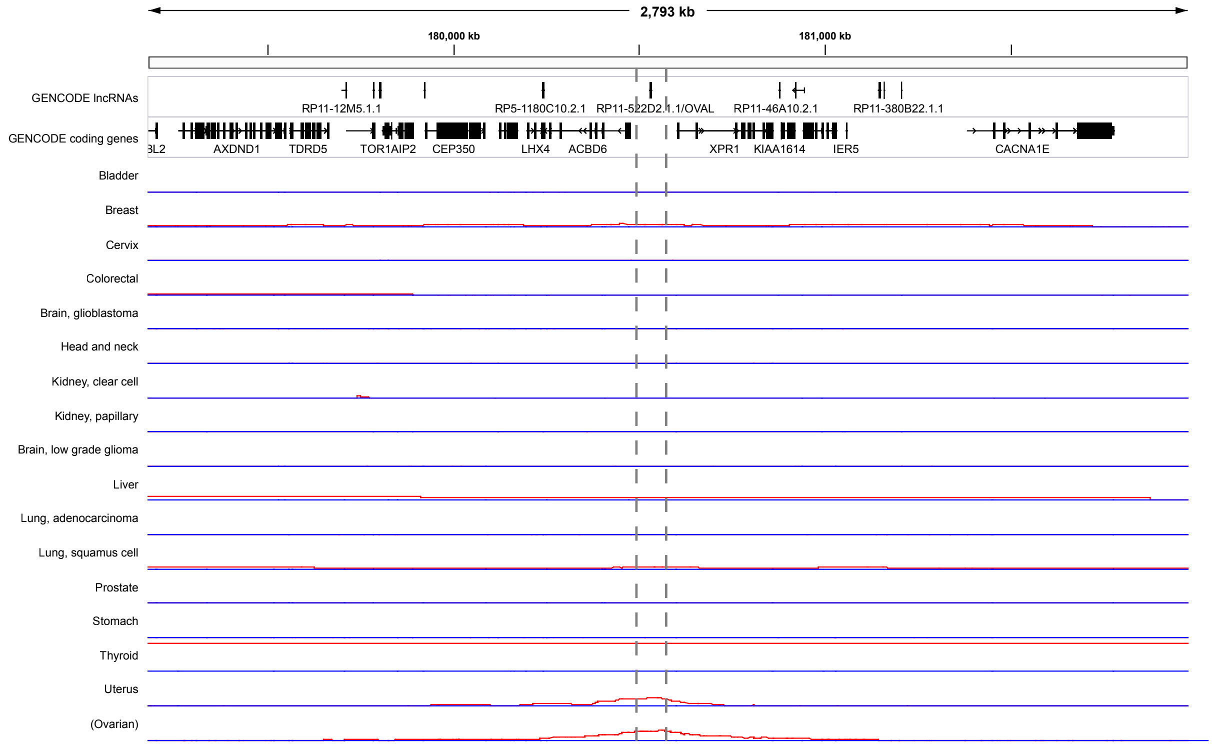

Supplementary Figure 6. Focal scores in the *OVAL* locus on chromosome 1 in 16 additional cancers. GISTIC scores for focal amplification/deletion were investigated in the *OVAL* locus. All TCGA cancers with at least 50 tumors at the time of download (Sep 2012, total 16) were considered. Genome coordinates in this figure refer to the Hg19 build. Endometrial tumors (Uterus) show a marked peak at the *OVAL* locus, similar to ovarian tumors (included for comparison).

Supporting tables

Differentiated

t-stat	GENCODE 11 gene ID	Symbol
4.14	ENSG00000225298.1	LINC00113
4.83	ENSG00000234142.1	RP11-276E17.2.1
4.20	ENSG00000240859.1	AC093627.10.1
4.33	ENSG00000258647.1	RP11-304N14.2.1
-4.20	ENSG00000226674.1	AC074093.1.1
-4.11	ENSG00000249685.1	RP11-360F5.3.1

Immunoreactive

t-stat	GENCODE 11 gene ID	Symbol
4.28	ENSG00000223387.2	RP11-408H1.3.1
4.54	ENSG00000227017.1	AC007036.6.1
4.11	ENSG00000231669.1	RP11-368D24__A.1.
4.62	ENSG00000232310.1	RP11-557H15.4.1
5.68	ENSG00000232756.1	RP5-1185I7.1.1
4.85	ENSG00000233535.1	RP1-122K4.2.1
5.50	ENSG00000235304.1	RP11-265P11.2.1
4.30	ENSG00000236389.1	RP1-287H17.1.1
4.47	ENSG00000236700.1	RP11-557H15.2.1
5.09	ENSG00000246084.2	CTD-2506J14.1.1
4.02	ENSG00000249096.1	RP11-290F5.1.1
4.44	ENSG00000253417.1	RP11-109J4.1.1
4.14	ENSG00000259641.1	RP11-279F6.3.1
-4.48	ENSG00000179219.5	LINC00311
-4.98	ENSG00000223403.2	AL132709.7.1
-4.04	ENSG00000224413.1	AP001476.2.1
-4.30	ENSG00000225746.3	AL132709.5.1
-4.15	ENSG00000231720.1	RP11-568A7.3.1
-4.57	ENSG00000238276.1	RP11-245J24.1.1
-4.04	ENSG00000248441.2	CTD-2536I1.1.1
-4.24	ENSG00000255983.1	RP11-1038A11.1.1
-4.11	ENSG00000257151.1	RP11-701H24.2.1
-4.21	ENSG00000260054.1	RP11-611L7.1.1
-4.16	ENSG00000260804.1	RP11-566E18.3.1
-4.08	ENSG00000261754.1	CTC-523E23.1.1

Mesenchymal

t-stat	GENCODE 11 gene ID	Symbol
4.41	ENSG00000100181.15	TPTEP1
8.58	ENSG00000223485.1	RP11-417E7.1.1
4.09	ENSG00000224514.1	AC093611.1.1
4.76	ENSG00000225383.1	SFTA1P
6.25	ENSG00000225783.2	MIAT
4.24	ENSG00000226791.2	AC109826.1.1
5.19	ENSG00000227082.1	AL592494.5.1
6.47	ENSG00000227579.1	RP1-35C21.2.1
13.19	ENSG00000229720.1	RP3-495K2.2.1
4.84	ENSG00000230499.1	AC108463.1.1
6.20	ENSG00000231133.1	HAR1B
4.65	ENSG00000232679.1	RP11-400N13.3.1
9.92	ENSG00000233521.1	RP5-1172A22.1.1
4.48	ENSG00000235565.1	RP11-86H7.7.1
5.37	ENSG00000235770.1	AC122136.1.1
4.86	ENSG00000235884.2	RP11-77I22.3.1
4.61	ENSG00000236819.1	AC087393.1.1
4.52	ENSG00000244758.1	AC093627.6.1
9.30	ENSG00000248870.1	CTD-2015A6.2.1
4.28	ENSG00000250334.1	RP11-234K19.2.1
5.39	ENSG00000253163.1	RP11-443C10.1.1
4.15	ENSG00000253688.1	RP11-567J20.2.1
7.45	ENSG00000253746.1	RP11-527N22.2.1
5.21	ENSG00000253983.1	RP1-16A9.1.1
7.28	ENSG00000254605.1	RP11-626H12.2.1
4.08	ENSG00000255191.1	RP11-626H12.1.1
9.76	ENSG00000259811.1	RP11-863P13.3.1
7.96	ENSG00000259847.1	RP11-95H3.1.1
4.98	ENSG00000260454.1	RP11-367F23.2.1
4.99	ENSG00000260578.1	CTD-2541J13.1.1
6.83	ENSG00000260868.1	RP11-394I13.1.1
9.87	ENSG00000261327.1	RP11-863P13.7.1
9.51	ENSG00000261742.1	RP11-256I9.1.1
-4.50	ENSG00000224843.1	LINC00240
-4.22	ENSG00000227544.1	AC018647.3.1
-4.14	ENSG00000233256.1	RP11-445K13.2.1
-4.56	ENSG00000234062.3	RP11-308D16.4.1
-4.13	ENSG00000235475.1	RP11-166O4.5.1
-4.53	ENSG00000239467.1	AC007405.6.1
-4.10	ENSG00000244345.1	RP11-654C22.2.1
-4.02	ENSG00000251381.2	CTC-497E21.4.1
-4.38	ENSG00000256084.1	RP11-134N1.2.1
-4.17	ENSG00000258946.1	RP11-58E21.4.1
-5.29	ENSG00000261455.1	RP5-1129J21.3.1

Proliferative

t-stat	GENCODE 11 gene ID	Symbol
4.32	ENSG00000174365.14	SNHG11
4.11	ENSG00000178248.10	AP000345.1.1
6.73	ENSG00000178947.7	LINC00086
4.89	ENSG00000179935.5	RP5-1068E13.3.1
4.99	ENSG00000203739.3	RP11-296O14.3.1
4.67	ENSG00000205611.3	RP4-610C12.4.1
5.82	ENSG00000205634.1	RP11-191L9.6.1
4.18	ENSG00000205673.1	AP000569.2.1
5.48	ENSG00000214039.3	RP11-474D1.3.1
5.40	ENSG00000215866.2	RP11-426L16.8.1
6.11	ENSG00000223393.1	RP5-858B6.3.1
4.88	ENSG00000223486.1	AC092198.1.1
5.03	ENSG00000223546.2	LL0XNC01-157D4.1.
4.10	ENSG00000223764.1	RP11-54O7.3.1
4.74	ENSG00000224167.1	RP3-522D1.1.1
4.45	ENSG00000225009.1	AC006509.6.1
5.27	ENSG00000225156.2	AC012354.6.1
4.40	ENSG00000225173.1	XXbac-BPG308K3.5.
4.56	ENSG00000225882.1	RP3-410B11.1.1
4.34	ENSG00000226965.1	AC003088.1.1
4.91	ENSG00000227060.1	AC004383.5.1
4.37	ENSG00000227165.1	RP11-323P17.1.1
4.35	ENSG00000227885.1	RP11-79N23.1.1
4.38	ENSG00000228271.1	RP11-513G11.2.1
5.10	ENSG00000228709.1	AP001065.15.1
4.13	ENSG00000228784.1	AC013400.2.1
5.40	ENSG00000229151.1	RP11-348F1.3.1
5.38	ENSG00000230294.3	RP13-507P19.2.1
4.46	ENSG00000230725.1	RP4-738P15.1.1
5.01	ENSG00000231327.1	AC016700.5.1
4.57	ENSG00000231772.1	RP1-154K9.2.1
4.00	ENSG00000231976.3	RP13-16H11.1.1
4.31	ENSG00000232006.1	AC005537.2.1
4.09	ENSG00000233396.1	RP11-458D21.1.1
4.40	ENSG00000233723.2	AC007092.1.1
4.10	ENSG00000233757.2	AC092835.2.1
4.66	ENSG00000233775.1	RP4-811H24.9.1
4.12	ENSG00000234261.1	RP11-146I2.1.1
4.45	ENSG00000234928.1	AP000344.3.1
7.07	ENSG00000235049.1	RP5-1096D14.2.1
4.82	ENSG00000236377.1	AC084809.3.1
4.27	ENSG00000236914.3	RP11-1008C21.2.1
6.06	ENSG00000236989.1	AC142119.1.1
4.42	ENSG00000237438.1	CECR7
4.25	ENSG00000237751.2	AC007040.5.1
4.16	ENSG00000238113.1	RP11-262H14.1.1
5.15	ENSG00000248740.1	RP11-328K4.1.1
4.40	ENSG00000249352.2	RP11-141O11.2.1
4.53	ENSG00000250519.2	RP11-680H20.2.1
4.04	ENSG00000251600.1	RP11-673E1.1.1
5.02	ENSG00000253361.1	RP11-675F6.3.1
4.89	ENSG00000256209.1	RP11-897M7.1.1
4.21	ENSG00000256232.1	RP11-771K4.1.1
7.12	ENSG00000257698.1	RP11-620J15.3.1
4.03	ENSG00000258131.1	RP11-541G9.1.1
4.40	ENSG00000258548.1	RP11-572M18.1.1
4.82	ENSG00000258592.1	RP11-108M12.3.1
5.18	ENSG00000260100.1	RP11-220I1.5.1
4.12	ENSG00000260163.1	RP11-521O16.2.1
4.34	ENSG00000260664.1	AC004158.3.1
-4.41	ENSG00000214049.3	UCA1
-4.86	ENSG00000224164.1	RP3-369A17.4.1
-5.72	ENSG00000224307.1	RP11-344B5.2.1
-4.71	ENSG00000228221.1	RP11-335L9.1.1
-4.91	ENSG00000229051.1	RP5-952N6.1.1
-4.34	ENSG00000233901.1	RP11-65J3.1.1
-5.53	ENSG00000237797.1	RP11-472N13.3.1
-4.15	ENSG00000238042.1	RP11-815M8.1.1
-5.52	ENSG00000239795.1	AC109826.2.1
-4.48	ENSG00000242147.1	RP13-463N16.6.1
-6.36	ENSG00000244459.2	RP11-1398P2.1.1
-4.82	ENSG00000245532.2	NEAT1
-4.49	ENSG00000248323.1	RP11-213H15.3.1
-4.21	ENSG00000248846.2	CTD-2016O11.1.1
-4.07	ENSG00000254288.1	RP11-6I2.3.1
-4.32	ENSG00000258689.1	RP6-65G23.1.1
-4.12	ENSG00000259225.2	RP11-1008C21.1.1

Supplementary Table 1. Intergenic lncRNAs that are transcriptionally induced (t-statistic > 4) or repressed (t-statistic < 4) exclusively in one of four previously defined expression subtypes.

Wide peak coordinates	Peak width	Type	Residual q	LncRNAs	Coding	LncRNAs in wide peak	Coding genes in wide peak
chr4:182346394-182458181	111787	Del	2.20E-31	1	0	RP11-665C14.2.1,	
chr1:178780208-178854243	74035	Amp	1.97E-02	1	0	RP11-522D2.1.1,	
chr3:170150840-170280412	129572	Amp	3.28E-56	1	0	RP11-152C17.1.1,	MECOM*,
chr6:57457872-58401277	943405	Del	1.07E-04	3	1	GUSBP4, XXbac-BPG55C20.7.1, RP11-343D24.2.1,	PRIM2,
chr17:21144429-21206026	61597	Del	2.68E-25	0	1		MAP2K3,
chr10:89701287-89711736	10449	Del	5.41E-11	0	1		PTEN,
chr17:26532345-26568338	35993	Del	6.58E-08	0	1		NF1,
chr16:77190685-77350206	159521	Del	1.15E-02	0	1		WWOX,
chr16:87965263-88055649	90386	Del	1.08E-02	0	1		ANKRD11,
chr3:117999463-118098130	98667	Del	7.18E-04	0	1		LSAMP,
chr2:141666036-141705031	38995	Del	2.39E-03	0	1		LRP1B,
chr19:34934008-35059410	125402	Amp	2.57E-96	0	1		CCNE1,
chr8:55315087-55564332	249245	Amp	1.30E-07	0	1		SOX17,
chr4:1699241-1763076	63835	Amp	1.65E-05	0	1		TACC3,
chr20:45338887-45497265	158378	Amp	9.04E-04	2	1	RP1-148H17.1.1, RP4-569M23.2.1,	ZMYND8,
chr11:120642837-120763395	120558	Amp	2.04E-02	0	1		SC5DL,
chr13:47887859-47909134	21275	Del	9.90E-16	0	2		LPAR6, RB1,
chr8:128754508-129388953	634445	Amp	3.70E-56	2	2	RP11-1136L8.1.1, PVT1,	MYC, TMEM75,
chr8:121118433-121381535	263102	Amp	4.58E-05	1	2	RP11-760H22.2.1,	DEPTOR, COL14A1,
chr18:22116085-22479962	363877	Amp	2.26E-03	0	2		KCTD1, TAF4B,
chr7:141684869-142210398	525529	Amp	3.47E-03	0	2		PRSS1, AC233282.2,
chr3:109082368-109306668	224300	Amp	4.89E-05	0	2		CD47, RP11-631B21.2.1,
chr20:29703246-29808748	105502	Amp	3.47E-04	1	2	RP11-243J16.7.1,	TPX2, BCL2L1,
chr22:49443563-49691432	247869	Del	1.01E-39	2	3	AC000036.4.1, AC002056.5.1,	RABL2B, ACR, SHANK3,
chr3:75428992-76088380	659388	Del	1.54E-03	1	3	RP11-413E6.8.1,	FRG2C, ROBO2, ZNF717,
chr6:107028442-107428188	399746	Amp	7.95E-03	2	3	RP1-60O19.2.1, RP1-60O19.1.1,	AIM1, RTN4IP1, QRSL1,
chr17:43923270-44005064	81794	Amp	2.82E-02	3	3	RP11-357H14.7.1, RP11-357H14.11.1, RP11-357H14.17.1,	HOXB3, HOXB1, HOXB2,
chr19:63736798-63811651	74853	Del	4.69E-05	0	4		MZF1, CHMP2A, UBE2M, TRIM28,
chr11:77509837-77679169	169332	Amp	9.28E-28	1	4	RP11-705O3.1.1,	GAB2, USP35, ALG8, KCTD21,
chr12:25059809-25394729	334920	Amp	2.66E-23	2	4	RP11-713N11.4.1, RP11-713N11.5.1,	CASC1, LRMP, KRAS, LYRM5,
chr1:36338557-36480225	141668	Amp	3.70E-03	0	4		TRAPPC3, THRAP3, MAP7D1, COL8A2,
chr12:44143268-45289633	1146365	Amp	3.38E-02	4	4	RP11-96H19.1.1, RP11-474P2.4.1, RP11-352M15.1.1, RP11-474P2.2.1,	SLC38A1, SLC38A2, SCAF11, ARID2,
chr10:78863997-79498631	634634	Amp	3.78E-05	2	5	RP13-39P12.3.1, RP11-126H7.3.1,	RPS24, POLR3A, DLG5, KCNMA1, AL391421.1,
chr22:28450357-28767355	316998	Amp	2.08E-04	1	5	CTA-85E5.10.1,	CABP7, ZMAT5, ASCC2, MTMR3, UQCR10,
chr17:24137681-24279608	141927	Amp	1.12E-02	0	5		PHF12, FLOT2, ERAL1, DHRS13, C17orf63,

** Wide peak has no coding overlaps, but is proximal to known oncogene MECOM/EVI1*

Supplementary Table 2. LncRNAs and coding genes intersecting with narrow focal regions identified by GISTIC (wide peak) in HGS-OvCa. Regions significant at $q < 0.05$ and encompassing at most 5 coding genes are included in the table. Coordinates refer to the Hg18 human genome assembly.

Rank	GENCODE 11 gene ID	Symbol	RPKM
1	ENSG00000258486.1	RN7SL1	8412.6
2	ENSG00000231486.3	AC096579.7.1	3613.8
3	ENSG00000251562.3	MALAT1	1617.2
4	ENSG00000175061.9	C17orf76-AS1	1216.5
5	ENSG00000260260.1	RP11-304L19.5.1	1128.7
6	ENSG00000177410.8	ZNFX1-AS1	666.7
7	ENSG00000234741.2	GAS5	571.3
8	ENSG00000258752.1	RP11-356K23.1.1	513.2
9	ENSG00000214049.3	UCA1	471.0
10	ENSG00000250742.1	RP11-834C11.4.1	448.8
11	ENSG00000226956.1	AP000432.2.1	406.5
12	ENSG00000245970.2	KB-1208A12.3.1	360.0
13	ENSG00000232388.1	LINC00493	359.0
14	ENSG00000245910.2	SNHG6	347.7
15	ENSG00000237232.2	ZNF295-AS1	278.9
16	ENSG00000247516.2	RP11-480D4.3.1	268.6
17	ENSG00000255135.1	RP11-111M22.3.1	256.7
18	ENSG00000237512.1	RP11-790G19.2.1	234.8
19	ENSG00000253518.1	CTD-2031P19.4.1	205.1
20	ENSG00000215808.2	RP11-371I1.2.1	200.4
21	ENSG00000225969.1	ABHD11-AS1	196.7
22	ENSG00000245532.2	NEAT1	183.1
23	ENSG00000196421.3	LINC00176	182.9
24	ENSG00000246067.2	RP11-113K21.5.1	181.2
25	ENSG00000261344.1	RP11-295M3.1.1	175.4
26	ENSG00000233901.1	RP11-65J3.1.1	171.8
27	ENSG00000228598.1	AC007001.4.1	169.7
28	ENSG00000246695.3	RP11-877E17.2.1	168.7
29	ENSG00000256234.1	RP11-283G6.4.1	168.6
30	ENSG00000255026.1	RP11-326C3.2.1	166.6
31	ENSG00000261122.1	RP11-352B15.1.1	162.2
32	ENSG00000260032.1	RP4-550H1.6.1	157.8
33	ENSG00000259439.1	RP11-89K21.1.1	157.4
34	ENSG00000259001.1	RPPH1.1	157.4
35	ENSG00000126005.10	MT1P3	157.4
36	ENSG00000258949.1	RP11-857B24.5.1	157.3
37	ENSG00000225792.1	AC004540.4.1	148.9
38	ENSG00000236081.1	AC074389.9.1	148.4
39	ENSG00000260054.1	RP11-611L7.1.1	148.2
40	ENSG00000257084.1	U47924.27.1	147.5
41	ENSG00000204272.6	RP11-622K12.1.1	144.2
42	ENSG00000233461.1	RP11-295G20.2.1	143.1
43	ENSG00000152931.6	PART1	133.7
44	ENSG00000240742.1	RP11-465L10.7.1	132.8
45	ENSG00000253196.1	RP11-706C16.7.1	131.3
46	ENSG00000255198.2	SNHG9	129.6
47	ENSG00000229847.2	EMX2OS	125.3
48	ENSG00000224259.1	RP11-48O20.4.1	123.6
49	ENSG00000260552.1	RP11-49I11.1.1	120.1
50	ENSG00000253716.1	RP13-582O9.5.1	116.9
51	ENSG00000247903.1	RP11-421F16.3.1	112.9
52	ENSG00000231106.1	AP000688.8.1	112.6
53	ENSG00000259943.1	RP1-39G22.7.1	111.9
54	ENSG00000256894.1	RP11-283G6.3.1	110.2
55	ENSG00000251129.1	RP11-734I18.1.1	106.8
56	ENSG00000261594.1	CTD-2562J17.3.1	104.6
57	ENSG00000255750.1	RP11-283G6.5.1	103.6
58	ENSG00000232335.1	RP11-435D7.3.1	101.2
59	ENSG00000225733.1	FGD5-AS1	99.5
60	ENSG00000212694.4	AC084018.1.1	98.0
61	ENSG00000204054.5	RP11-492E3.1.1	97.8
62	ENSG00000233016.1	SNHG7	96.9
63	ENSG00000227121.1	RP11-319F12.2.1	95.2
64	ENSG00000261183.1	RP11-532F12.5.1	94.9
65	ENSG00000225138.2	CTD-2228K2.7.1	94.6
66	ENSG00000232677.1	AC092296.1.1	94.6
67	ENSG00000254615.1	RP11-395G23.3.1	94.5
68	ENSG00000163364.4	AC017048.3.1	92.9
69	ENSG00000236618.1	AC100748.2.1	91.8
70	ENSG00000255717.1	SNHG1	91.0
71	ENSG00000228262.1	AC073218.2.1	89.4
72	ENSG00000214548.8	MEG3	88.1
73	ENSG00000254337.1	RP11-865I6.2.1	86.5
74	ENSG00000236719.2	RP11-522D2.1.1	86.4
75	ENSG00000229807.3	XIST	86.1
76	ENSG00000254682.1	RP11-660L16.2.1	84.9
77	ENSG00000248538.2	RP11-10A14.5.1	83.7
78	ENSG00000226363.2	AC009336.24.1	82.5
79	ENSG00000167912.5	RP11-25K19.1.1	81.0
80	ENSG00000206337.6	HCP5	79.0
81	ENSG00000226197.1	RP11-536O18.1.1	78.5
82	ENSG00000225484.2	RP11-773D16.1.1	77.9
83	ENSG00000258616.1	RP11-369C8.1.1	77.7
84	ENSG00000255509.1	RP11-445F12.1.1	77.0
85	ENSG00000257588.1	RP11-469H8.6.1	76.9
86	ENSG00000231295.1	RP4-797C5.2.1	72.4
87	ENSG00000233975.1	RP11-288L9.1.1	72.0
88	ENSG00000256916.1	RP11-817J15.2.1	71.3
89	ENSG00000260708.1	CTA-29F11.1.1	70.0
90	ENSG00000228288.2	RP11-480I12.3.1	69.1
91	ENSG00000239467.1	AC007405.6.1	68.5
92	ENSG00000234694.1	RP1-92O14.3.1	68.3
93	ENSG00000249859.2	PVT1	67.7
94	ENSG00000248275.1	CTC-338M12.3.1	67.3
95	ENSG00000229941.1	AC012499.1.1	67.0
96	ENSG00000232533.1	AC093673.5.1	66.5
97	ENSG00000236546.1	RP1-118J21.5.1	66.4
98	ENSG00000237513.1	RP11-325F22.2.1	66.1
99	ENSG00000236318.1	AC019117.1.1	65.6
100	ENSG00000224189.2	AC009336.23.1	65.3

Supplementary Table 3. GENCODE lncRNAs ranked by maximum expression in 407 TCGA ovarian tumors as determined by RNA-seq.
